# Surgical hands antisepsis with alcohol-based preparations: cost-effectiveness, compliance of professionals and ecological benefits in the Brazilian healthcare scenario

**DOI:** 10.1186/2047-2994-4-S1-P162

**Published:** 2015-06-16

**Authors:** ME Graf, A Machado, LL Mensor, D Zampieri, R Campos

**Affiliations:** 1 Infectious Diseases and Infection Control, Hospital UniversitárioCajuru PUCPR, Curitiba, Brazil; 2 Infectious Diseases and Infection Control, BeneficênciaHospitais, Brazil; 3 Laboratórios Braun, Brazil

## Introduction

Surgical hands disinfection with alcohol-based handrub preparation effectively removes Gram-positive and Gram-negative organisms (including multi-resistant ones), as well as fungi and viruses. Evidence allows concluding that alcohol-based handrub preparation can replace the alternatives traditionally applied such as chlorhexidine and promote reductions in associated costs, including indirect ones such as water supply and brushes disposal.

## Objectives

To assess the cost-effectiveness of surgical hands disinfection technique with alcohol-based handrub preparation versus scrubbing with chlorhexidine under the perspective of Brazilian hospitals.

## Methods

Cost-effectiveness analysis through a decision model by comparing the two techniques for surgical hands disinfection:

a) Use of alcohol-based handrub preparation (Softalind® Pure, B. Braun Medical AG),

b) Scrubbing with chlorhexidine brushes. Outcomes considered were reduction of microbial counting (clinical scenario) and water savings (ecological scenario).

Economic outcomes were direct medical costs and indirect costs (water consumption)

## Results

Total costs of the technique with Softalind® Pure was 46% lower than the costs of the technique with chlorhexidine brushes. Additionally, the clinical scenario has shown superior effectiveness for the alcohol-based handrub preparation, due to the higher in vitro microbial counting of 23% than its comparator In the ecological scenario, the reduction of 18,5 liters of water per procedure with the use of alcohol-based handrub preparation generates cost savings besides the saving in the water consumption itself.

## Conclusion

The present evaluation pointed out several advantages for the use of alcohol-based handrub preparation for surgical hands disinfection. Among them the significant reduction in microbial counting, improvements in compliance of professionals due to less time for preparation (1 minute for alcohol-based preparations vs. 3 minutes for scrubbing with chlorhexidine) and less irritant effect under the skin, besides great savings in costs and water consumption and brushes disposal.

## Disclosure of interest

M. E. Graf Consultant for: BBraun, A. Machado: None declared, L. Mensor: None declared, D. Zampieri: None declared, R. Campos: None declared.

